# Flipping the switch: dynamic modulation of membrane transporter activity in bacteria

**DOI:** 10.1099/mic.0.001412

**Published:** 2023-11-10

**Authors:** Rory Elston, Christopher Mulligan, Gavin H. Thomas

**Affiliations:** ^1^​ Department of Biology, University of York, York, UK; ^2^​ School of Biosciences, University of Kent, Kent, UK

**Keywords:** ABC, bacteria, membrane transport, post-translational regulation

## Abstract

The controlled entry and expulsion of small molecules across the bacterial cytoplasmic membrane is essential for efficient cell growth and cellular homeostasis. While much is known about the transcriptional regulation of genes encoding transporters, less is understood about how transporter activity is modulated once the protein is functional in the membrane, a potentially more rapid and dynamic level of control. In this review, we bring together literature from the bacterial transport community exemplifying the extensive and diverse mechanisms that have evolved to rapidly modulate transporter function, predominantly by switching activity off. This includes small molecule feedback, inhibition by interaction with small peptides, regulation through binding larger signal transduction proteins and, finally, the emerging area of controlled proteolysis. Many of these examples have been discovered in the context of metal transport, which has to finely balance active accumulation of elements that are essential for growth but can also quickly become toxic if intracellular homeostasis is not tightly controlled. Consistent with this, these transporters appear to be regulated at multiple levels. Finally, we find common regulatory themes, most often through the fusion of additional regulatory domains to transporters, which suggest the potential for even more widespread regulation of transporter activity in biology.

## Introduction

The transport of small molecules across the bacterial cell membrane is essential for cellular growth. Many key nutrients, ions and co-factors are impermeable to the bacterial cytoplasmic membrane and require specialized transport proteins to facilitate their uptake. The accumulation of some small molecules and ions can be deleterious to cell growth, so their transport across the membrane is tightly regulated. While genetic control of levels of transport protein in cells is well known – take, for example, expression of the lactose permease, LacY, encoded as part of the canonical *lac* operon [1, 2] – transport can also be effectively regulated by direct post-synthesis modulation of transport protein activity by small molecule or protein–protein interactions. Understanding these regulatory mechanisms will provide insight into the adaptations bacteria undergo to survive under various conditions and may reveal targets for antimicrobial drug development.

Transport proteins are integral membrane proteins that are capable of passive or active transport across the membrane. Passive transporters, otherwise known as facilitators, allow the transport of a substrate down a concentration gradient, a process that does not require energy input. Active transporters, on the other hand, facilitate the energetically unfavourable movement of a substrate *against* its gradient by harnessing an energy source. Active transporters can be broadly divided into two groups, depending on their energy source: primary transporters, which use a primary source of energy, for example, ATP hydrolysis; and secondary transporters that utilize electrochemical gradients across the membrane, specifically H^+^ and Na^+^ gradients, to power transport. While many transporters are involved in uptake of chemicals, many others are involved in export and efflux, for example for initial export of antibiotics [3] and then subsequent resistance to them [4]. For example, of the 68 predicted ATP binding cassette (ABC) transporters in *Escherichia coli* K-12, 57 are involved in uptake and 11 in export [5].

Regardless of the energy required to power transport, active transporters are dynamic proteins that undergo multiple, often large-scale, conformational changes to move the substrate from one side of the membrane to the other. Transporter mechanisms are minimally composed of an inward-facing state (IFS), in which the substrate-binding site is exposed to the cytoplasmic side of the membrane, and an outward-facing state (OFS), where the substrate-binding site is accessible to the extracytoplasmic space (periplasm or extracellular milieu). Therefore, transport activity can be controlled by modulating one or more of these stages of the transport process.

The ability to rapidly ‘switch off’ a transporter, i.e. inhibit the transport cycle, could be advantageous under changing environmental conditions. If a bacterial cell is using a transporter to enable growth, then a reduction in its transcription and translation, combined with general turnover of the existing protein in the membrane, might not be fast enough for the cell to adapt to new conditions effectively. For example, if an essential but toxic metal becomes concentrated above safe levels through transporter activity, then slow removal of the transporter from the membrane might not be a fast enough response to ensure homeostasis. With the above-described general transport cycle in mind, transport activity could be terminated rapidly by interfering with substrate binding through competitive inhibition, preventing substrate interactions via allosteric inhibition, cutting off the energy source, or preventing the essential conformational changes.

While there are numerous studies of transporters themselves acting as sensors as part of signal transduction systems to control gene expression [6], a good example being the *E. coli* Mlc protein that senses flux through the glucose phosphotransferase system [7], less is known about how transporter activity is regulated once they are functioning in the membrane. Here, we use selected examples to describe the major types of regulation of activity known for bacterial transporters, namely the use of small molecules, small peptides and larger signal transduction proteins for rapid, often reversible control before then covering recent discoveries around targeted proteolysis of transporters for more permanent inactivation.

## Small molecule regulation of transporter activity

It has long been established that the activity of bacterial transporters can be regulated by the concentration of intracellular metabolites [8, 9]. In many cases, the uptake of nutrients is regulated by a process referred to as transinhibition, where the transporter is inhibited by its own translocated substrate upon it reaching a threshold cytoplasmic concentration; a process akin to feedback inhibition experienced by some soluble enzymes. In other cases, regulation can be mediated by another intracellular metabolite, an early example being glucose inhibition of fructose uptake in *E. coli *, which was termed ‘catabolite inhibition’ [10]. In this first section, we discuss the structural basis of small molecule protein-level regulation of transporters from multiple structurally distinct families, which often requires additional small regulatory domains to provide the added allosteric feedback properties of the protein.

### ATP-binding cassette (ABC) importers regulated by substrate-dependent transinhibition

The molecular basis for transinhibition was first revealed for two ABC transporters, the *E. coli* MetNI methionine transporter [11] and the *Methanosarcina acetivorans* ModABC molybdate/tungstate transporter [12]. Briefly, ABC transporters (TC 3 .A.1 [13]) are a large superfamily of primary-active transporters responsible for the uptake and extrusion of a multitude of ions and compounds across bacterial membranes [14]. They are defined by the presence of a highly conserved nucleotide-binding domain (NBD) that binds and hydrolyses ATP. The conformations induced by the binding and hydrolysis of ATP are coupled to the transmembrane domain (TMD), which undergoes substantial isomerization. As the membrane spanning TMD houses a substrate binding site, these coupled conformational changes facilitate the alternating access of the substrate-binding site to both sides of the membrane. While ABC transporters involved in efflux from the cell are usually composed of a TMD and an NBD, ABC importers in bacteria often employ a substrate-binding protein (SBP) that resides in the periplasm or is lipid-anchored to the outer leaflet of the cytoplasmic membrane [15]. A canonical ABC uptake mechanism consists of the SBP that binds substrate with high affinity and selectivity, which docks with the TMD to trigger the ATP-dependent transport cycle described above.

**Fig. 1.**
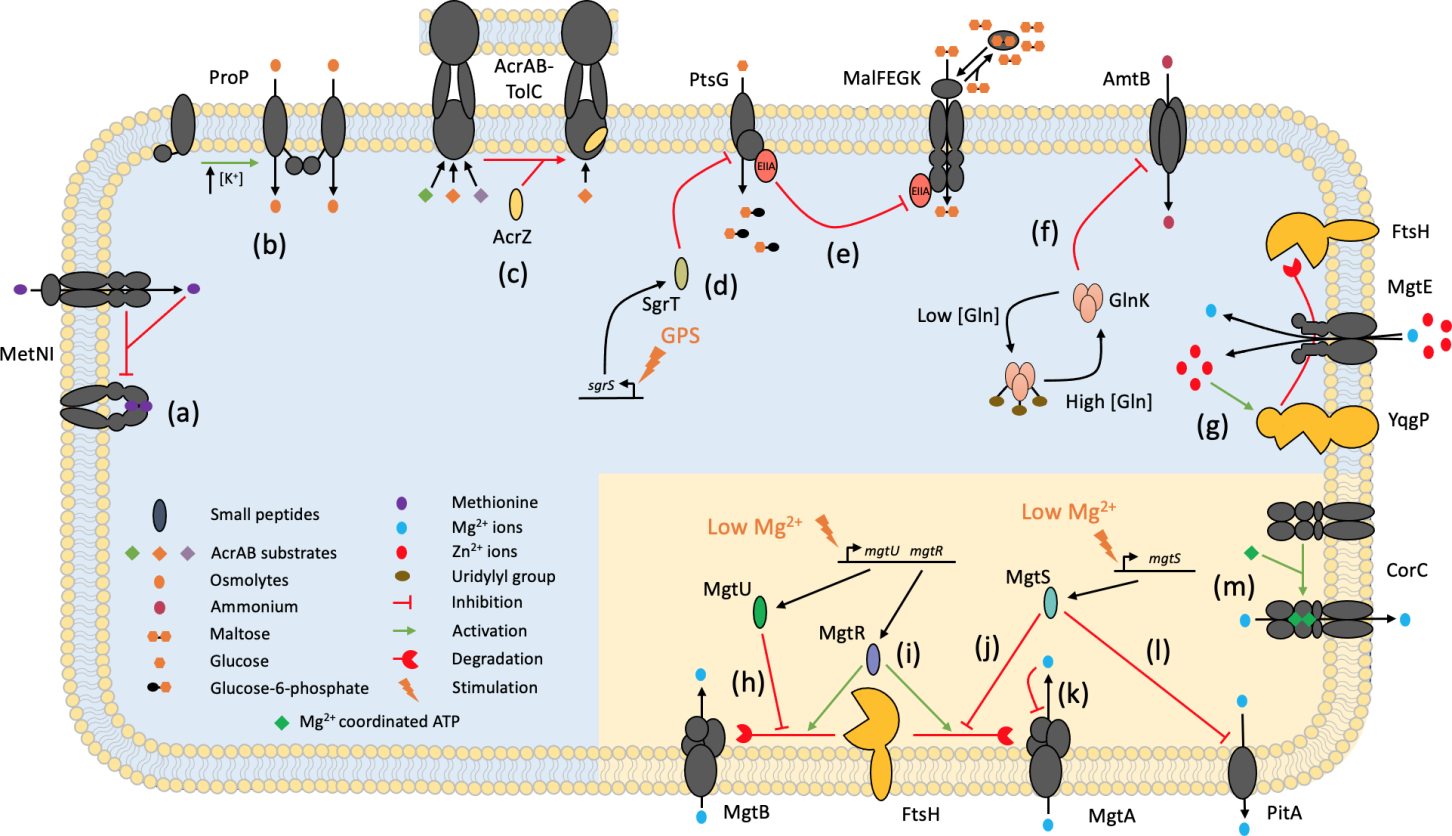
An overview of the various post-translational regulatory mechanisms used by bacteria to control transport of metabolites. Representative examples of the various regulatory mechanisms described in the main text are shown with a blue background. A collection of examples of post-translational regulation of Mg^2+^ transport in bacteria is shown with a yellow background.

**Fig. 2.**
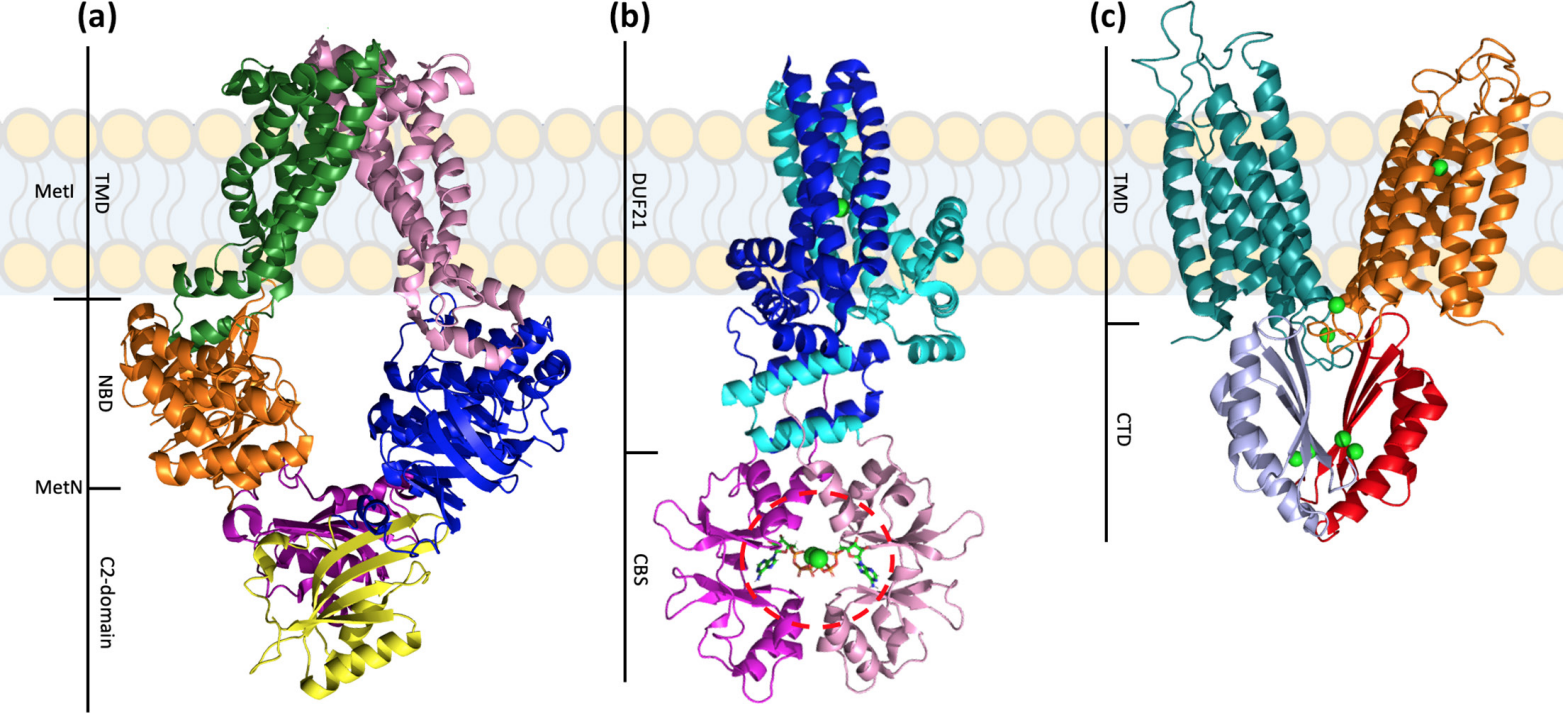
Crystal structures of selected transporters regulated through small-molecule binding to cytoplasmic regulatory domains. (**a**) Crystal structure of the MetNI transporter showing the transmembrane domains (TMDs, green, pink), nucleotide-binding domains (NBDs, orange, blue) and the regulatory C2 domains (purple, yellow). Adapted from [11]. (**b**) A crystal structure of the homodimeric MtCorB without the C-terminal CorC domain showing the DUF21 transmembrane domains (dark blue, light blue) and regulatory cystathionine-β-synthase domains (CBS, pink, magenta). Mg^2+^ ions are shown as green spheres. The 2 Mg^2+^-ATP molecules bound to the CBS domains are indicated by the red circle. Adapted from [37]. (**c**) A crystal structure of the YiiP transporter showing the TMDs (green, orange) and C-terminal domains (CTDs, grey, red). Zn^2+^ ions are shown as green spheres. Adapted from [43]. All figures were rendered using PyMOL using PDB codes 3DHW, 7M1T and 3H90.

Transinhibition of methionine uptake was one of the earliest regulatory mechanisms discovered for transporters, being described by Robert Kadner in 1975, with the observation that *E. coli* cells pre-loaded with methionine exhibited substantially lower rates of transport than untreated cells [9]. The transporter responsible, the dl-methionine uptake system, MetNIQ (otherwise known as MetD), has two copies of the NBD, MetN, two copies of the transmembrane protein, MetI, and the cognate SBP, MetQ, which has a high affinity for both l- and d-methionine [16, 17]. The crystal structure of the MetNI complex was captured in the inward-facing state with the ATP-free NBDs splayed apart [11] (Fig. 1a, Fig. 2a). The NBDs contain the typical ATP-binding domain found in other ABC transporters but also an additional C-terminal domain, termed the C2-domain, which dimerizes to bridge the two NBDs [11]. This dimerization is facilitated by the binding of two methionine molecules sandwiched at the interface of this auxiliary domain, which prop open the NBDs, thus preventing ATP hydrolysis and inhibiting transport [11, 18, 19]. A similar mechanism is observed in the molybdate/tungstate ABC transporter ModABC, from *M. acetivorans *, where the NBD (ModC) contains a similarly positioned C-terminal regulatory domain (although a different fold to the MetNI C2 domain) [12] (see Table 1 for summaries of systems not illustrated in Fig. 1). Here, molybdate and tungstate bind at the dimeric interface of the C-terminal domains, preventing the transporter from cycling between its inward- and outward-facing states and thus inhibiting further transport [12].

**Table 1.** Examples of the transporter regulation mentioned in this review

Type of regulation	Type of transport	Transporter family (TCDB code)	Transporter name	Organism(s)	Regulator	Substrate class	Regulatory mechanism	Evidence
Small molecule	1 ° active transport	ATP-binding cassette (3 .A.1)	MetNI	* Escherichia coli *	Methionine	Amino acids	Restriction of conformational flexibility	S, B [11, 18, 19]
Small molecule	1 ° active transport	ATP-binding cassette (3 .A.1)	ModABC	* Methanosarcina acetivorans *	Molybdate /tungstate	Inorganic ions	Restriction of conformational flexibility	S [12]
Small molecule	Uniport	CorA metal ion transporter (MIT) (1 .A.35)	CorA	* E. coli *	Mg^2+^	Metal ions	Conformational changes result in pore closure	S, B [26, 27]
Small molecule	1 ° active transport	ATP-binding cassette (3 .A.1)	MgtA	* E. coli *	Mg^2+^	Metal ions	Unknown	B [31]
Small molecule	2 ° active transport	(CNNM)/CorB (1 .A.112.)	CorB	* Methanoculleus thermophilus *	ATP, Mg^2+^	Nucleotides, metal ions	Binding of ATP and Mg^2+^ activates transporter	S [37]
Small molecule	2 ° active transport	(CNNM)/CorB (1 .A.112.)	CorC	*Thermus parvatiensis*	ATP, Mg^2+^	Nucleotides, metal ions	Binding of ATP and Mg^2+^ activates transporter	S [38]
Small molecule	2 ° active transport	Cation diffusion facilitator (2 .A.4)	YiiP	* E. coli *	Zn^2+^	Metal ions	Binding of Zn^2+^ to CTD causes transport-activating conformational changes	S, B [40–43, 45]
Small molecule	2 ° active transport	Cation diffusion facilitator (2 .A.4)	YiiP	* Shewanella oneidensis *	Zn^2+^	Metal ions	Binding of Zn^2+^ to CTD causes transport-activating conformational changes	S [44, 45, 50]
Small molecule	2 ° active transport	Cation diffusion facilitator (2 .A.4)	CzcD	* Ralstonia metallidurans *	Zn^2+^	Metal ions	Binding of Zn^2+^ to CTD activates transporter	B [46]
Small molecule	2 ° active transport	Cation diffusion facilitator (2 .A.4)	ZitB	* E. coli *	Zn^2+^	Metal ions	Binding of Zn^2+^ to CTD activates transporter	B [46]
Small molecule	2 ° active transport	Cation diffusion facilitator (2 .A.4)	CzrB	* Thermotoga maritima *	Zn^2+^	Metal ions	Binding of Zn^2+^ to CTD activates transporter	S, B [47]
Small molecule	2 ° active transport	Cation diffusion facilitator (2 .A.4)	MamM	* Magnetospirillum gryphiswaldense *	Zn^2+^	Metal ions	Binding of Zn^2+^ to CTD activates transporter	S, B [48, 49]
Osmoregulation	2 ° active transport	Major facilitator superfamily (**2 .A.1**)	ProP	* E. coli *	Increase in environmental osmolarity	Osmolytes	Dimerization of CTD activates transporter	B [61, 67]
Osmoregulation	2 ° active transport	Betaine/carnitine/choline transporter (2 .A.15)	BetP	* Corynebacterium glutamicum *	Increase in environmental osmolarity	Osmolytes	CTD-mediated regulation	[68, 69]
Osmoregulation	1 ° active transport	ATP-binding cassette (3 .A.1)	OpuA	* Lactococcus lactis *	Increase in environmental osmolarity	Osmolytes	Osmolarity is sensed through an HTH motif	S, B [70]
Small protein	1 ° active transport	The PTS glucose-glucoside (Glc) family (4 .A.1)	PtsG	* E. coli *	SgrT	Monosaccharides	Binding-dependent inhibition of transport	B, G [79, 82]
Small protein	2 ° active transport	The inorganic phosphate transporter (PiT) family (2 .A.20)	PitA	* E. coli *	MgtS	Metal ions	Thought to inhibit transport	G [83]
Small protein	1 ° active transport	The P-type ATPase (P-ATPase) superfamily (3 .A.	MgtA	* Salmonella enterica* serovar Typhimurium	MgtS	Metal ions	Protects transporter from FtsH-mediated degradation	B [100]
Small protein	1 ° active transport	The MntP Mn^2+^ exporter (MntP) family (2 .A.107)	MntP	* E. coli *	MntS	Metal ions	Thought to inhibit transport	G [84, 85]
Small protein	1 ° active transport	The resistance–nodulation–cell division (RND) superfamily (2 .A.6)	AcrAB–TolC	* E. coli *	AcrZ	Small lipophilic molecules	Modulates transporter substrate specificity	B, G [86, 87, 89, 90]
Small protein	1 ° active transport	The resistance–nodulation–cell division (RND) superfamily (2 .A.6)	WatABO	* Methylomonas* sp*.* DH-1	WatS1	Small organic acids	Modulates transporter substrate specificity	G [92]
Small protein	1 ° active transport	The P-type ATPase (P-ATPase) superfamily (3 .A.3)	MgtA	* S. enterica* serovar Typhimurium	MgtR	Metal ions	Promotes FtsH-mediated degradation of transporter	B, G [95, 96]
Small protein	1 ° active transport	The P-type ATPase (P-ATPase) superfamily (3 .A.3)	MgtB	* S. enterica* serovar Typhimurium	MgtR	Metal ions	Promotes FtsH-mediated degradation of transporter	B, G [95, 96]
Small protein	1 ° active transport	The P-type ATPase (P-ATPase) superfamily (3 .A.3)	MgtB	* S. enterica* serovar Typhimurium	MgtU	Metal ions	Protects transporter from FtsH-mediated degradation	B, G [96]
Small protein	1 ° active transport	The ferrous iron uptake (FeoB) family (9 .A.8)	FeoB	*S. enterica,* * Vibrio cholerae *	FeoC	Metal ions	Protects transporter from FtsH-mediated degradation	B, G [104, 106, 107]
Cytoplasmic signal transduction proteins	2 ° active transport	The ammonium channel transporter (Amt) family (1 .A.11)	AmtB	* E. coli *	GlnK (P_II_ family)	Inorganic ions	T-loop insertion blocks translocation channel	S, B, G [112–115]
Cytoplasmic signal transduction proteins	2 ° active transport	The ammonium channel transporter (Amt) family (1 .A.11)	Amt1	* Synechocystis* sp*.* strain PCC 6803	P_II_	Inorganic ions	T-loop insertion likely blocks translocation channel	B, G [116, 117]
Cytoplasmic signal transduction proteins	1 ° active transport	ATP-binding cassette (3 .A.1)	NrtABCB	* Synechocystis* sp*.* strain PCC 6803	GlnB (P_II_ family)	Nitrate/nitrite	Phosphorylated P_II_ protein binding inhibits transport	B, G [118–120]
Cytoplasmic signal transduction proteins	1 ° active transport	ATP-binding cassette (3 .A.1)	UrtABCDE	* Synechocystis* sp*.* strain PCC 6803	GlnB (P_II_ family)	Urea	Phosphorylated P_II_ protein binding inhibits transport	B, G [116]
Cytoplasmic signal transduction proteins	2 ° active transport	Sodium solute symporter (SSS)	SbtA	* Synechocystis* sp*.* strain PCC 6803	SbtB (P_II_ family)	Organic ion	AMP-bound P_II_ protein binding inhibits transport	S, B, G [122, 123]
Crosstalk between transporters	2 ° active transport	The PTS glucose-glucoside (Glc) family (4 .A.1)	LacY	* E. coli *	MFS	Short oligosaccharides	Loop binding inhibits substrate-binding step	S, B [127, 128]
Crosstalk between transporters	1 ° active transport	ATP-binding cassette (3 .A.1)	MalFEGK	* E. coli *	EIIA^Glc^	Short oligosaccharides	NBD binding prevents ATP hydrolysis	S, B [129, 130]
Crosstalk between transporters	2 ° active transport	The glycoside–pentoside–hexuronide (GPH) : cation symporter family (2 .A.2)	LacS	Streptococci, * Lactobacillus *	HPr	Short oligosaccharides	Phosphorylation of EIIA^Glc^-like domain inhibits transport	B, G [131–133]
Rhomboid protease-based regulation	Uniport	The Mg^2+^ transporter-E (MgtE) family (1 .A.26)	MgtE	* Bacillus subtilis *	YqgP	Metal ions	Mg^2+^-stimulated cleavage of transporter+priming for FtsH-mediated degradation	B, G [144, 145]

Evidence codes are indicated using the following general data types: S, structural, G, genetic; B, biochemical.

### Direct substrate-dependent regulation is widespread in diverse metal uptake and efflux systems

The metal-dependent transinhibition described in the previous section for ModABC is only one example of this phenomenon observed in metal transporters. The use of metals by bacteria is often a double-edged sword as, although many divalent metals, including Zn^2+^, Fe^2+^, Ni^2+^ and Cu^2+^, are essential as cofactors for metalloenyzmes, they are also toxic to the cell at elevated cytoplasmic levels, hence, there has been strong evolutionary selection to maintain these concentrations in the useful but tolerated range [20–22]. In this section, we illustrate this concept by highlighting examples of direct substrate-mediated transport regulation, including both uptake and efflux systems, for Mg^2+^ and other divalent cations.

The importance of Mg^2+^ for the function of many enzymes [23] has ensured that bacterial cells maintain intracellular concentrations of ~30 mM, with a free cytosolic concentration of ~0.3–1 mM [24]. To keep the intracellular concentration high enough, bacteria have evolved multiple routes for uptake that function under different external Mg^2+^ concentrations. Under non-limiting Mg^2+^ conditions, bacteria such as *E. coli* and *Bacillus subtilis* use channel proteins, CorA and MgtE (Fig. 1g), respectively, to facilitate uptake of Mg^2+^ ions. Given the cationic nature of the substrate, this process is powered by both the inwardly directed Mg^2+^ gradient and the negative-inside membrane potential [25]. Although not a transporter per se, it is notable that CorA can be gated by Mg^2+^ binding to a regulatory domain, leading to pore closure when the internal concentration of Mg^2+^ is too high [26, 27]. When Mg^2+^ is more limiting, bacteria use active transporters, such as the P-type ATPase MgtA, that couple ATP hydrolysis to Mg^2+^ uptake [28], powering Mg^2+^ transport against its gradient or an unfavourable membrane potential [29]. MgtA consists of a nucleotide-binding domain (N), an actuator domain (A), a phosphorylation domain (P) and a transmembrane region that forms the pathways across the membrane [30]. Together, these components undergo a series of complex conformational changes in response to ATP binding, substrate binding and autophosphorylation events to facilitate vectorial substrate transport [30]. While most other P-type ATPases are relatively insensitive to Mg^2+^ concentrations and can maintain transport activity in Mg^2+^ concentrations exceeding 20 mM, MgtA is only active at the much lower concentration of ~1 µM Mg^2+^ with strong inhibition exhibited at concentrations over 1 mM (Fig. 1k) [31]. While the molecular mechanism of inhibition has not yet been elucidated, the biochemical data fit nicely with the known cytosolic concentration of free Mg^2+^ [32, 33], suggesting that this is a physiologically relevant process.

While switching off uptake systems can rapidly halt further accumulation of Mg^2+^ if the metal exceeds a tolerable cytoplasmic concentration, bacteria also encode active metal efflux pumps, allowing them to grow in environments with high Mg^2+^ levels. For example, *Salmonella enterica* serovar Typhimurium (*S*. Typhimurium) can tolerate ~300 mM Mg^2+^, whereas *Staphylococcus aureus* is able to handle ~800 mM Mg^2+^ in the medium [34, 35]. The prodigious tolerance exhibited by *S. aureus* is due to the Mg^2+^ efflux pump, MpfA, which, if disrupted, leads to growth inhibition in concentrations of as little as ~10 mM external Mg^2+^ [35]. MpfA belongs to the CBS-pair domain divalent cation transport mediators (CNNM)/CorB transporter family (TC# 1.A.112) [36] that consist of a DUF21 transmembrane domain and a regulatory cytosolic cystathionine-β-synthase (CBS) pair domain, which is capable of binding Mg^2+^-ATP. The structures of two CNNM/CorB family members, CorB from *Methanoculleus thermophilus* (MtCorB) [37] and CorC from *Thermus parvatiensis* (TpCorC) [38], has shed light on some details of the transport and regulatory mechanisms (Fig. 2b). Both proteins form similar homodimeric complexes with a Mg^2+^-binding site in the centre of the transmembrane DUF21 domain, demarcating the Mg^2+^ translocation pathway [37, 38]. The contacts between the cytosolic CBS pair domains are mediated by two ATP molecules bound at the interface, in an arrangement reminiscent of the NBDs in ABC transporters [39]. However, unlike in an ABC transporter, the bound ATP is not hydrolysed. Instead, in combination with Mg^2+^, ATP binding triggers adoption of the active conformational state of the pump [37], which then uses the transmembrane Na^+^ electrochemical gradient to power transport [37] (Fig. 1m). Therefore, in contrast to the previous examples in this section that are negatively regulated, in this case, Mg^2+^-ATP is acting as a trans*activator* of efflux activity.

Efflux of other toxic divalent cations can be catalysed by the cation diffusion facilitator (CDF) family of efflux proteins (TC# 2 .A.4), which are proton-driven antiporters [40]. The first structurally characterized CDF transporter was YiiP (FieF) from *E. coli* (Fig. 2c), which revealed a homodimeric arrangement, with each protomer consisting of a transmembrane domain, each with an independent Zn^2+^ translocation pathway, and a large cytoplasmic C-terminal domain (CTD) that adopts a metallochaperone-like fold [41, 42]. The EcYiiP structure revealed the presence of three well-conserved Zn^2+^-binding sites: one in the membrane embedded domain; one at the interface of the transmembrane domains and one in the CTD at the dimer interface [42, 43]. Comparison of outward- and inward-facing structures of EcYiiP and SoYiiP from *Shewanella oneidensis* suggests that alternating access to each membrane-embedded binding site is achieved through relative rocking and rotating of a bundle of four transmembrane helices [44, 45].

The Zn^2+^-binding sites in the CTDs are distant from the translocation pathway and are thought to play a role in positively regulating efflux activity. The CTD interface is stabilized by the coordination of four Zn^2+^ ions in EcYiiP and mutagenesis of even a single Zn^2+^-binding site residue in the CTD substantially reduces transport activity [42, 43, 45]. In addition, complete removal of the CTD from other CDF transporters considerably reduces transport activity [46]. Förster resonance energy transfer (FRET) measurements with full-length EcYiiP reveal Zn^2+^-dependent closure of the CTDs, which is not observed in a Zn^2+^-binding site mutant [43]. Furthermore, metal-dependent CTD closure has been observed for isolated CTDs taken from CzrB from *Thermotoga maritima* and MamM from *Magnetospirillum gryphiswaldense* using protein crystallography, SAXS and PELDOR, further supporting the predicted regulatory mechanism of this domain [47–49]. Collectively, these data suggest that in the presence of low cytoplasmic Zn^2+^ concentrations, the CTD is in its *apo* state and the transporter adopts a state of low activity. Then, upon an increase in the Zn^2+^ concentration, the Zn^2+^-bound CTDs associate, driving the membrane domains into an active state, providing another example of a transactivation process. However, cryo-EM structures of SoYiiP suggest that only small conformational changes occur in the CTD between the *apo* and metal bound states [50], suggesting that perhaps large conformation changes driven by the regulatory domains are not required in every case.

### Osmoregulation of transport activity

Bacterial osmoregulation refers to a set of processes enacted by bacterial cells in response to changes in the osmolarity of the external environment [51–53]. One way in which this regulation is achieved is through the action of osmosensory transporters, a group of importers whose activity is increased in response to hyperosmotic conditions so as to accumulate compatible solutes to prevent dehydration of the cell. Extensive work from numerous laboratories has provided mechanistic insight into how these transporters are regulated, primarily through the study of three systems that represent different transport families, namely, ProP [MFS (TC# 2 .A.1)], BetP [betaine-carnitine transporter (BCCT) family (TC# 2 .A.15)] and OpuA (ABC), which have been reviewed comprehensively elsewhere [51–53]. Studies of the different transporters reconstituted in proteoliposomes have identified trends in the stimuli to which the transporters respond under conditions of hyperosmotic stress, which reflect the *in vivo* response of the cells. Generally, the activity of these proteins is activated by increasing concentrations of inorganic cations on the cytoplasmic side of the membrane (such as K^+^, Na^+^ and Li^+^) [54–56] and the presence of molecular crowding agents (such as polyethylene glycol (PEG)) [55, 57]. Activation is also dependent on lipid composition, with anionic lipids increasing the osmolarity required for activation of all three transporters [58–60].

Mechanistically, early work on *E. coli* ProP identified the role of an extended cytoplasmic C-terminal domain (CTD) [61] as being important for osmoregulation, a feature later found in BetP and OpuA, although not sequence related [62–64]. Altering or removing this CTD results in changes in transporter sensitivity to osmotic stimuli, suggesting that such domains play an important role in osmosensory behaviour [57, 65, 66]. Current models of the regulatory mechanism suggest that under normal physiological conditions the CTD associates with the membrane, which locks ProP in an inward-facing state, preventing transport [61]. It has been proposed that the lowering of membrane order that occurs due to increasing K^+^ concentrations during hyperosmotic conditions results in loss of association between residues of the ProP CTD and *E. coli* membrane lipids [67]. Release of the CTD allows it to form a coiled-coil with a CTD from a neighbouring ProP, forming a homodimer, which together then activates both ProP transporters (Fig. 1b). Similarly, homotrimeric BetP contains three long CTDs (one on each protomer) that undergo cytoplasmic K^+^-dependent changes in its interactions, switching it between binding to either the membrane, intracellular loops of the same protomer and/or the N-terminus of the neighbouring protomer. However, the precise details of these transitions and the mechanistic consequences remain to be determined [68, 69].

The ABC transporter OpuA employs two different mechanisms to rapidly alter its activity [70], both through the introduction of additional sequence features in the NBD. The first is a unique membrane proximal helix–turn–helix (HTH) motif containing a series of positively charged residues that modulate the sensitivity of the transporter to osmotic change. The NBD also contains tandem CBS domains, similar to that mentioned for MpfA, although here they bind cyclic di-AMP, which acts as an ‘override switch’ to deactivate the transporter [70]. Together, these two regulatory mechanisms provide a rapid route to modulate transporter activity, with the cyclic-di-AMP acting as a ‘double brake’ to stop hyper accumulation of compatible solutes, which is in itself also detrimental to bacterial viability [70]. Interestingly, the KUP family of K^+^ transporters can also be deactivated by binding of cyclic-di-AMP to its CTD, which in this case is a phosphopantetheine adenylyltransferase (PPAT) domain rather than a CBS domain [71].

## Modulation of transport activity by small regulatory proteins

Small proteins, generally defined as being <50 aa in length, are being increasingly recognized as regulatory components in multiple bacterial processes [72–74], which has been the topic of multiple comprehensive reviews [73–77]. Here, we focus on a selection of examples of small proteins that regulate bacterial transporter function by either direct interaction or as adaptors/modifiers that target transporters for degradation.

### Direct modulation of activity through small protein binding

An excellent example of a small protein with a regulatory role in metabolite transport was discovered during studies of the response of *E. coli* to glucose phosphate stress (GPS), which occurs upon accumulation of toxic levels of cytoplasmic sugar phosphates [78]. GPS stimulates the transcription of the *sgrS* gene, which encodes a small RNA (sRNA), SgrS, that consists of two distinct sections [79]. The 3′ end of the sRNA base pairs to and prevents the translation of the *manXYZ* and *ptsG* mRNA, which encode the components of the mannose- and glucose-specific phosphotransferase systems (PTSs), respectively, thus reducing any further production of intracellular sugar phosphates [80, 81]. In contrast, the 5′ end of *sgrS* encodes the 43 aa peptide SgrT [79], which binds to and inhibits the activity of the glucose-specific PTS protein PtsG (Fig. 1d) [82]. The precise molecular details of this peptide-mediated inhibition are not yet clear, but an AlphaFold model of SgrT (UniProt ID: C1P5Z7) predicts an alpha-helical hairpin that could plausibly interact with the membrane-bound transporter. Ectopic expression of just the 3′ or 5′ ends of the *sgrS* gene in a Δ*sgrS* background results in a stark difference in uptake of a radiolabelled glucose analogue, with SgrT completely inhibiting uptake whilst SgrS has a minimal effect [82]. This highlights the importance of post-translational regulators in enacting rapid responses to conditions that are toxic to bacteria. Two further examples of small proteins that inhibit transport activity in *E. coli* are MgtS (31 aa) and MntS (42 aa), which regulate the Mg^2+^-coordinated phosphate-exporter PitA (Fig. 1l) and the Mn^2+^-exporter MntS, respectively, to prevent loss of the metal ions under depleted conditions [83–85].

A different example of small protein-based regulation mediated through transporter binding is AcrZ, a small *E. coli* protein (49 aa) that interacts with the AcrAB–TolC RND (TC# 2.A.6) efflux pump by forming a single-helical ‘belt’ around the transmembrane domains of AcrB [86–88]. The absence of AcrZ reduces the specificity of the RND pump, a modulation of activity that also appears to be related to binding of the lipid cardiolipin to the AcrBZ (Fig. 1c) [89, 90]. While the interaction between AcrZ and AcrB has been characterized extensively through structural work [86, 88, 89], it is not currently clear how the function of AcrZ is itself modulated, which would be an important requirement for any dynamic regulatory element. We note that *E. coli* contains at least one other similar small protein, YajC, that interacts with AcrAB–TolC to modulate its activity. Interestingly, YajC binds to AcrB in a similar site to AcrZ [91], suggesting potential competition between these modulators.

The small protein, WatS1 (55 aa), from *Methylomonas* sp. DH-1 can also bind and regulate the activity of an RND pump to alter its substrate specificity quite specifically [92] . The RND pump WatABO is required for acid stress survival and ordinarily recognizes and exports a range of small organics [92]. However, upon binding WatS1, WatABO specifically increases its ability to efflux acetate, but not other tested organic acids [92]. In addition, a recent structure of a novel ABC transporter for lipid uptake from *Mycobacterium tuberculosis* revealed a previously unknown but conserved small transmembrane protein, LucB, that was proposed to be involved in regulating activity [93]. Overall, these data suggest that small proteins can play important roles in modulating the activities of various types of transporter.

### Small protein binding to direct transporter turnover

The general turnover of membrane proteins in bacteria is a relatively poorly understood process, but the membrane-anchored AAA+protein FtsH, an essential protein in *E. coli *, is known to play a role in this process. When originally discovered, FtsH was found to be involved in the removal of misfolded integral membrane proteins [94]. Since this time, and as will be described herein, FtsH is now known to also function in the specific degradation of properly folded, functional transporters, which is a drastic but effective route for downregulating transporter activity [95–97].

A prime exemplar of this regulatory mechanism is the interplay between the small proteins MgtR (30 aa) and MgtU (28 aa), which are both involved in cytoplasmic Mg^2+^ homeostasis in *S*. Typhimurium [95, 96]. Under Mg^2+^-depleted conditions, MgtR and MgtU are produced alongside the P-type ATPase Mg^2+^ importer, MgtB [96–99]. While MgtR binds to both MgtB and a second P-type ATPase, MgtA, to promote their FtsH-dependent degradation, MgtU only binds and stabilizes MgtB, specifically protecting MgtB from FtsH-mediated proteolysis (Fig. 1h,i) [95–97]. Interestingly, a similar regulatory interaction is present in *E. coli *, where the small protein MgtS, which is noted in the previous section to inhibit the activity of PitA, can also bind to and stabilize MgtA (Fig. 1j) [100]. The ultimate consequence of MgtR-based small protein-mediated regulation in *S*. Typhimurium is the differential retention of MgtB over MgtA under prolonged Mg^2+^ depletion. Yeom *et al*. speculate that this difference in abundance of the closely related transporters MgtA and MgtB is linked to the fact that MgtB, unlike MgtA, is required for long-term survival of *Salmonella* in low-Mg^2+^ environments [101], such as within macrophages [96]. In accordance with this, MgtU was demonstrated to promote survival of *Salmonella* inside mouse macrophages, presumably through increasing MgtB stability. MgtB displays greater Mg^2+^ affinity than MgtA, as well as a decreased propensity for inhibition, reasons posited to enable the transporter to aid *Salmonella* infection of macrophages [96, 102].

Another excellent example of transport regulation via small protein-directed proteolysis is FeoC, which influences the activity of FeoB, the primary bacterial importer of ferrous (Fe^2+^) iron for many bacteria under anaerobic conditions [103–105]. In *Vibrio cholerae *, no FeoB-dependent uptake of Fe^2+^ is observed in the absence of FeoC [104]. In addition, FeoB abundance in *Salmonella enterica* is substantially diminished in the absence of the *feoC* gene, suggesting that the lack of observed Fe^2+^ transport in *V. cholerae* is due to FeoB turnover [104, 105]. FeoB depletion in the *S. enterica* Δ*feoC* strain is prevented when FtsH protease levels are depleted, suggesting that loss of FeoB activity is through FtsH-dependent proteolysis. Interestingly, when cells adapt from anaerobic (conditions under which FeoB is produced) to aerobic conditions the FeoC levels in *S. enterica* drop rapidly due to O_2_-induced damage from an iron–sulfur (Fe–S) cluster in FeoC, accelerating its degradation by Lon protease (an AAA+ protease for soluble proteins) [106, 107]. Therefore, an elegant model has been proposed in which FeoC turnover under aerobic conditions leads to the subsequent proteolysis of FeoB by FtsH [106], which facilitates rapid modulation of transport activity based on an environmental signal.

## Regulation of activity by cytoplasmic signal transduction proteins

Beyond the binding of small peptides to transporters, there are also multiple examples of larger soluble proteins that can interact with transporters on their cytoplasmic surface to regulate their activity. One of the best understood examples of this comes from the need for tight control of the flux of ammonium during growth of *E. coli *. During nitrogen limitation, *E. coli* induces expression of the ammonium transporter, AmtB, which aids the ATP-dependent glutamine synthetase (GS) enzyme in nitrogen assimilation [108, 109]. The *amtB* gene was noted to be genetically linked to *glnK*, which encodes a P_II_ family signal transduction protein [109, 110]; both GlnK and AmtB form trimeric complexes, which are key to their ability to interact in a 1 : 1 ratio [111].

When bacteria pre-adapted to nitrogen limitation encounter a pulse of free ammonia, they need to quickly inhibit the energy-hungry ATP-dependent route for nitrogen assimilation. This inhibition is achieved by downregulation of GS, but also via GlnK directly plugging the translocation pathway of AmtB, preventing further uptake of ammonium (Fig. 1f) [112, 113]. The GlnK–AmtB interaction is itself regulated; the tight interaction required for inhibition of AmtB is only possible following the removal of a uridylyl group from GlnK, which is triggered by the action of upstream regulatory proteins [112, 114, 115]. Interestingly, this same P_II_ protein-mediated regulation has been observed for the AmtB equivalent in cyanobacteria, Amt1, except that the interaction in this case is controlled by reversible phosphorylation rather than uridylylation, potentially providing more dynamic control of transport [116, 117].

P_II_ family signal transduction proteins have also been shown to directly modulate the activity of other families of transporters. In cyanobacteria, the P_II_ protein GlnB can interact with the nucleotide-binding domains of the nitrate/nitrite ABC transporter, NrtABCD, and the urea ABC transporter, UrtABCDE, to inhibit their uptake activity [118–121]. While the molecular details of these interactions remain to be elucidated, it is interesting to note that inhibition of these two ABC transporters is facilitated by interactions with different sites on the P_II_ proteins, suggesting that individual P_II_ proteins could have multiple targets. A P_II_ family protein, SbtB, has also been shown to directly regulate the *Synechocystis* PCC 6803 bicarbonate transporter, SbtA, which is a member of the Na^+^ solute symporter (SSS) transporter family (TCDB #2 .A.21) [122]. As with the GlnK–AmtB interaction, the trimeric SbtB interacts directly with the trimeric SbtA to lock the transporter into a single conformational state, thus inhibiting its function. In this case, the interaction only occurs when SbtB is bound to AMP, adding an extra layer of regulation to this inhibition [123]. In conclusion, P_II_ proteins appear to have evolved to interact with multiple transporter types to regulate their activity, although their use does appear to be restricted to transporters that can function as trimers.

## Regulation by crosstalk from other transporters

As bacteria switch from using one nutrient to another, changes in transcription lead to downregulated expression of now redundant transporters and catabolic pathways, and replacement with new ones. In *E. coli *, there is a strong hierarchy for the use of different sugars, with glucose being preferred [124]. In parallel with transcriptional control, there are additional levels of regulation that ensure that glucose is taken up first even in the presence of other sugars. The key player in this process is the bacterial phosphoenolpyruvate : sugar phosphotransferase system(s) [PTS(s)], one of which is the primary route for glucose uptake in *E. coli* [125]. PTSs consist of a membrane-embedded sugar permease and a series of cytoplasmic components that collectively uptake and concomitantly phosphorylate molecules of sugar or sugar derivatives [125, 126].

One of these cytoplasmic components, the EIIA^Glc^ protein, forms part of the phosphorelay system that powers the PTS. EIIA^Glc^ can exist in either a phosphorylated or dephosphorylated form, with the dephosphorylated form predominant under conditions where glucose uptake is active [124]. To favour glucose uptake, dephosphorylated EIIA^Glc^ can also bind to and inhibit a range of non-PTS transporters, preventing uptake of other sugars and preventing induction of genes encoding unnecessary catabolic proteins [125]. An excellent example of this regulation is found in *E. coli *, where dephosphorylated EIIA^Glc^ associates with the cytoplasmic loops of lactose permease, LacY, an MFS transporter [127], reducing its ability to bind its substrates [128]. In addition, the same dephosphorylated EIIA^Glc^ interacts with nucleotide-binding domains (MalK) of the maltose ABC transporter, MalFEGK (Fig. 1e) [129]. Structural analysis of the inhibitory complex reveals that the binding of two dephosphorylated EIIA^Glc^ to dimeric MalK prevents the interdomain rotations required for ATP hydrolysis during the transport cycle, thus inhibiting function [130]. This process is also highly reversible, as if glucose transport slows or stops, phosphorylated EIIA^Glc^ accumulates, which then removes inhibition of the other sugar transporters.

An interesting variation of this concept is in the streptococci and lactobacilli, where a EIIA^Glc^-like domain is fused directly to the lactose/galactose antiporter, LacS [131]. Here, the phosphocarrier HPr can phosphorylate the EIIA^Glc^-like domain to modulate the activity of transport in response to the metabolic needs of the cell [132, 133] and thus PTS-mediated regulation ensures that lactose is not taken up when glucose is available. LacS represents the sole example of bacterial transporter regulation being achieved through direct phosphorylation of the transport protein in question. This apparent scarcity of phosphorylation-mediated control contrasts strongly with eukaryotes, in which it is a well-characterized mode of regulation [134]. A notable example of such regulation in eukaryotes is the serotonin transporter of mammals, for which phosphorylation by various kinases affects protein levels at the plasma membrane [135].

## Rhomboid proteases: emerging roles in controlled membrane protein turnover

We have seen earlier in this review that small proteins can function to regulate transport by either providing protection from or stimulation of degradation by the general AAA+ protease, FtsH, introducing the idea of engaging the general pathway for membrane protein turnover to modulate transporter activity. There is increasing evidence now for additional functions for membrane-bound proteases in transporter regulation and targeted membrane protein turnover more generally. While the leader (or signal) peptidase, SPase I, is well studied and will act on membrane transporters, this is still only a membrane-anchored protease domain that acts on the membrane surface [136]; however, the rhomboid protease family, which we discuss in this final section, comprises examples of truly integral membrane-bound enzymes, that have solved the rather paradoxical problem of catalysing a hydrolytic (water requiring) reaction in a membrane environment where water is explicitly excluded.

The rhomboid proteases were discovered approximately 20 years ago in eukaryotes, playing a role in the cleavage of EGFR ligand precursors in *Drosophila melanogaster* [137]. These receptor substrates are typical of the type of proteins cleaved by eukaryotic rhomboid proteases, in that they are generally single-pass transmembrane proteins, i.e. membrane-anchored proteins, where the protease action is required for release from the membrane in a controlled way. Soon after the first discovery of rhomboid it was noted that rhomboids were widespread in bacteria [138] and that different bacterial homologues, including an *E. coli* protein called GlpG, were able to catalyse the same cleavage as the *Drosophila* rhomboid-1 when expressed in mammalian cells [138]. This remarkable observation supported a widely conserved mechanism for substrate recognition and enzymatic cleavage in these enzymes and further studies on the function and mechanism of GlpG were completed [139], leading to its structure being solved in 2006 [140]. This work confirmed a six-transmembrane helix organization with a Ser–His dyad as the catalytic centre and a large periplasmic cavity open to water that is required for the catalytic cycle.

Another bacterial protein shown to rescue the *Drosophila* rhomboid-1 phenotype was a mysterious protein called AarA, whose function was uncovered through the work of Philip Rather and his group [141]. Briefly, they had discovered a mutant in the *Enterobacteriaceae *, *Providencia stuartii *, that failed to secrete a membrane-anchored quorum-sensing molecule [142]. After mapping multiple mutants with this phenotype to the same gene, *aarA*, which encoded a rhomboid-like protein, they were able to demonstrate that AarA cleaves a unique N-terminal extension of the TatA protein, an essential component of the Tat protein secretion system, the removal of which by AarA is required for TatA function and interaction with TatC [143]. Since this pioneering work was completed, another rhomboid protein, YqgP, from *B. subtilis *, was discovered in 2020 to directly modulate magnesium uptake. YqgP can cleave the previously described magnesium transporter, MgtE, between its first and second transmembrane regions, thus removing the CBS domain and inactivating transport function (Fig. 1g) [144]. This cleavage only occurred in the cells during conditions where uncontrolled MgtE activity would be detrimental, supporting the idea that this is a regulatory mechanism. In fact, the authors showed that an additional N-terminal domain in the rhomboid protein was responsible for sensing the metals when at toxic levels and presumably activating the ability of the rhomboid domain to cleave MgtE. Finally, they also demonstrated that YqgP has an additional role in directing the MgtE to FtsH for further degradation, a function that surprisingly does not depend on the catalytic activity of the rhomboid domain [145], but which is in fact a phenomenon already known from some other eukaryotic rhomboid proteins such as derlins [144]. These exciting findings reveals increasingly complex layers of regulation of cellular processes in bacterial cells, in this case following their initial discovery in eukaryotes.

## Concluding remarks

We have outlined a diverse range of systems where transporter activity changes rapidly in the cell, with a common theme being rapid reduction of net uptake, by either reducing uptake, promoting efflux, or triggering protein inactivation and/or degradation. While transinhibition was described conceptually over 50 years ago, molecular insight has come from biochemistry and structural biology to allow us to understand these mechanisms, which from the examples outlined in this review, often require additional small fused domains that are required for this ‘plug on’ addition of allosteric control. With this knowledge one can browse completed genomes of bacteria and find many examples of transporters that have additional fused domains where the function is not known, suggesting that there is extensive use of regulatory domains in transporter biology. We recently assessed the extent of these adaptations for the MFS transporter family and found many classes of fused domains, usually at the C-terminus of the protein, that have possible regulatory functions [146, 147].

One domain we have seen in multiple guises in this review is the cystathionine β-synthase (CBS) domain. This domain has been utilized by many different families of transporters to allow allosteric control by some form of nucleotide and is usually found as tandem repeat on the C-terminus. One more complex variation of this is the function of the CBS domain in the MgtE Mg^2+^ channel [148]. Here, ATP binding to the domain is prerequisite for Mg^2+^ binding to the same domain, which is required for channel closing, meaning that the domain is now integrating two signals [149]. The CBS domains are also found fused to MFS transporters, which supports more generally the idea of ‘plug and play’ domains that can be recruited by diverse transporters to add regulatory properties. MFS transporters are also found fused to cyclic nucleotide-binding domains (CNBDs), PAS domains and UspA fusions [146, 147], suggesting that there is a library of different regulatory domains that can be recruited. The UspA fusions are interesting in that they have been observed in many cases, either directly fused or encoded within the transporter operon [150], but none have yet been experimentally characterized in the context of transporter regulation. We have suggested that, given that these domains are known to bind ATP, this might be another novel way to regulate transporter activity based on the cellular energy status [147], but this is just one example of where there are exciting opportunities to discover new functions for bacterial proteins.

We should also expect to find increasing numbers of examples of small integral membrane protein regulators of transporters, which were for a time totally overlooked, because bacterial genome annotation tools would not even recognize them as genuine coding sequences [74]. Partly through their discovery as ‘additional density’ in X-ray and cryo-EM structures of transporters, a number of these peptides have been recognized and in some cases their regulatory/modulatory functions described. More widely, the ‘dark proteome’ of small proteins is being recognized as having a multiplicity of function in both bacteria and eukaryotes [151].

The exciting new evidence for controlled protein degradation is an emerging field for bacteria, following behind earlier work in eukaryotes. Perhaps these systems have more subtle roles than they play in eukaryotes, as they often do not appear to be essential genes. An example comes from recent work on the Rhom7 protein from *Shigella sonnei* [152]. Here, deletion of both Rhom7 and the GlpG rhomboids resulted in no obvious growth-related phenotypes, but by careful analysis the authors discovered that the proteases appear to target ‘orphan’ subunits of membrane protein complexes found in the membrane and so perhaps have a more general role in membrane protein quality control. Emerging evidence from other organisms such as *Brucella abortus* and *Corynebacterium glutamicum* shows that removal of rhomboid proteins alters levels of multiple membrane proteins, including some solute transporters [153, 154]. Together, these data suggest that rhomboid proteins are used widely in bacteria, but seemingly for fine tuning of overall membrane functions, although there is clearly much more to learn about their biological roles in other bacterial systems.

What other regulatory mechanisms might be out there waiting to be discovered? We have seen how small molecules can bind to transporters and that other proteins can alter their activity, but not yet considered their physical environment within a lipid bilayer. There is evidence for the activity of some of the osmoregulatory transporters mentioned in this review, like BetP, requiring negatively charged phosphatidyl glycerol (PG) lipids for their activation by osmotic stress [155], and there is increasing evidence that the nature of the lipids interacting with the transporter can alter its conformational landscape [156, 157]. If there were dynamic ways to alter the lipid composition in the cell, through relocalizing transporters in the membrane, for example, or altering their interactions with their surrounding lipids, then one could imagine that these might be additional ways to alter transporter function on a relatively short timescale [158].

In conclusion, we have drawn together literature highlighting diverse examples of transporter regulation (Fig. 1, Table 1). The journey of a membrane transporter from the initial transcription of the gene, translation of protein and its insertion into the membrane are regulated processes, but only form short periods in the transporter’s lifetime, and it is not surprising that multiple additional routes have evolved in biology to control its activity once active in the membrane. Application of this information could have multiple uses in actively modulating cellular function. Some will serve as excellent drug targets, but the tendency for bacteria to build redundancy into their small molecule transporters means that in many cases they will not be suitable targets [159]; however, their manipulation during biotechnological processes to limit the flow of a nutrient and/or product in or out of the cell is certainly possible, although to date examples of ‘transporter engineering’ sit around changing transporter profiles through manipulating gene expression rather than protein activity [160–162]. We hope this review serves as a useful summary of what is known and act as a catalyst for more study in this area.
